# The safety and efficacy of finasteride for transgender men with androgenetic alopecia: a case series

**DOI:** 10.1186/s13256-025-05562-y

**Published:** 2025-09-29

**Authors:** Yusuke Tominaga, Tomoko Kobayashi, Yuko Matsumoto, Tomoko Sako, Takatoshi Moriwake, Satoshi Horii, Takuya Sadahira, Satoshi Katayama, Takehiro Iwata, Shingo Nishimura, Kensuke Bekku, Kohei Edamura, Masami Watanabe, Motoo Araki

**Affiliations:** 1 Department of Urology, Dentistry and Pharmaceutical Sciences, Okayama University Graduate School of Medicine, 2-5-1, Shikata-Chou, Kita-Ku, Okayama, 700-8558 Japan; 2 Department of Urology, Good Life Hospital, Hiroshima, Japan; 3 Department of Urology, Hiroshima City Hiroshima Citizens Hospital, Hiroshima, Japan; 4 Center for Innovative Clinical Medicine, Dentistry and Pharmaceutical Sciences, Okayama University Graduate School of Medicine, Okayama, Japan

**Keywords:** Finasteride, Dihydrotestosterone, Transgender men, Androgenetic alopecia, Resumption of menstruation

## Abstract

**Background:**

Testosterone replacement therapy is commonly used in transgender men for masculinization. One of the most common adverse effects of testosterone replacement therapy is androgenetic alopecia. In Japan, finasteride is approved exclusively for cisgender men and is not indicated for transgender men. The aim of this clinical trial was to evaluate the safety and efficacy of finasteride in transgender men with androgenetic alopecia.

**Case presentation:**

This study included three transgender men (assigned female at birth, identifying as male), aged 44, 43, and 29 years. All participants were of Asian ethnicity. A clinical trial was conducted from October 2021 to December 2023. Transgender men aged 20–60 years who had not undergone hysterectomy, were undergoing testosterone replacement therapy, and who had been diagnosed with stage ≥ II androgenetic alopecia on the basis of the Norwood–Hamilton scale were recruited. The participants initiated treatment with 0.2 mg of finasteride per day for 3 months (phase 1). If no adverse events above grade 2 occurred, the dose was increased to 1.0 mg per day for an additional 3 months (phase 2). The primary endpoints were the incidence of treatment-related adverse events at 1 week, 1 month, and 3 months, as well as the rate of participants continuing treatment at 3 months. None of the patients experienced serious adverse events at 3 months, and all the patients extended their treatment to a total of 6 months. Improvements of at least one stage on the N–H scale were observed, but two participants experienced resumption of menstruation.

**Conclusion:**

Finasteride appears to be a safe and effective treatment for androgenetic alopecia in transgender men undergoing testosterone replacement therapy. However, its potential for reducing some of the effects of testosterone replacement therapy warrants further investigation. Trial registration: jRCT, jRCTs061210040, registered 7 October 2021, https://jrct.mhlw.go.jp/latest-detail/jRCTs061210040.

**Supplementary Information:**

The online version contains supplementary material available at 10.1186/s13256-025-05562-y.

## Background

Transgender and gender-diverse (TGD) individuals may seek gender-affirming medical and/or surgical treatments to align their bodies with their gender identities [1]. Psychiatric counseling remains the first-line approach; however, if gender dysphoria persists, medical transition may be considered. According to the Japanese guidelines for the diagnosis and treatment of gender incongruence, either hormone therapy or gender-affirming surgery can be initiated first; however, hormone therapy is frequently the initial step [2].

For transgender men (those who are assigned female at birth but who identify as male), testosterone replacement therapy (TRT) achieves physical masculinization. The effects of TRT are expected to include cessation of menstruation, atrophy of internal genitalia, voice deepening, and masculinization of the body; however, adverse events such as polycythemia, dyslipidemia, hyperuricemia, and androgenetic alopecia (AGA) may occur [3–5].

Dihydrotestosterone (DHT) is considered a major causative agent of AGA. Testosterone is converted into DHT by the enzyme 5-alpha-reductase type 2; finasteride acts to improve hair loss by inhibiting this enzyme, thereby suppressing DHT levels [6]. The efficacy and safety of finasteride have been reported for cisgender men with AGA, and the medication has been approved by the Pharmaceutical Affairs Bureau in Japan for delaying the progression of male-pattern alopecia [7].

However, finasteride is not authorized in Japan for use in transgender men who have not undergone hysterectomy/oophorectomy and who are officially registered as female, despite its potential utility, and there are few reports of finasteride use in transgender men. A retrospective study in Spain examined its safety and efficacy, but the doses, administration routes, and formulations used in the study are not approved in Japan [8].

Transgender men experiencing AGA as a result of TRT need new treatment options. If finasteride proves effective for this population, it would mark a significant advancement in their care. Our goal is to expand the limited body of knowledge in this area by evaluating and confirming the safety of finasteride for transgender men treated at our institution.

We recruited transgender men who underwent TRT at our department at Okayama University Hospital between November 2021 and December 2023. The inclusion criteria were patient age between 20 and 60 years at the time of study consent, receipt of TRT from our department, prehysterectomy status, and AGA stage II or higher according to the Norwood‒Hamilton (N–H) scale. The exclusion criteria included a history of allergy to finasteride, the presence of liver dysfunction, and a diagnosis of a severe mental disorder (e.g., severe depression). Additionally, investigators were able to use their discretion to exclude individuals who were deemed inappropriate for the study.

The primary endpoints of this study were the incidence of treatment-related adverse events at 1 week, 1 month, and 3 months from the start of finasteride administration and the percentage of participants who continued treatment at 3 months. The secondary endpoints included the treatment effect, which was assessed as improvement from baseline via photographs of the parietal area and classified according to the N–H scale [9]. Additional evaluations included blood testing, physical examinations, and assessments of subjective symptoms. Body mass index (BMI), body fat percentage, and muscle mass were measured via the “male” setting on the Tanita Inner Scan 50, model BC-303 (Tanita Corporation, Japan).

**Fig. 1:**
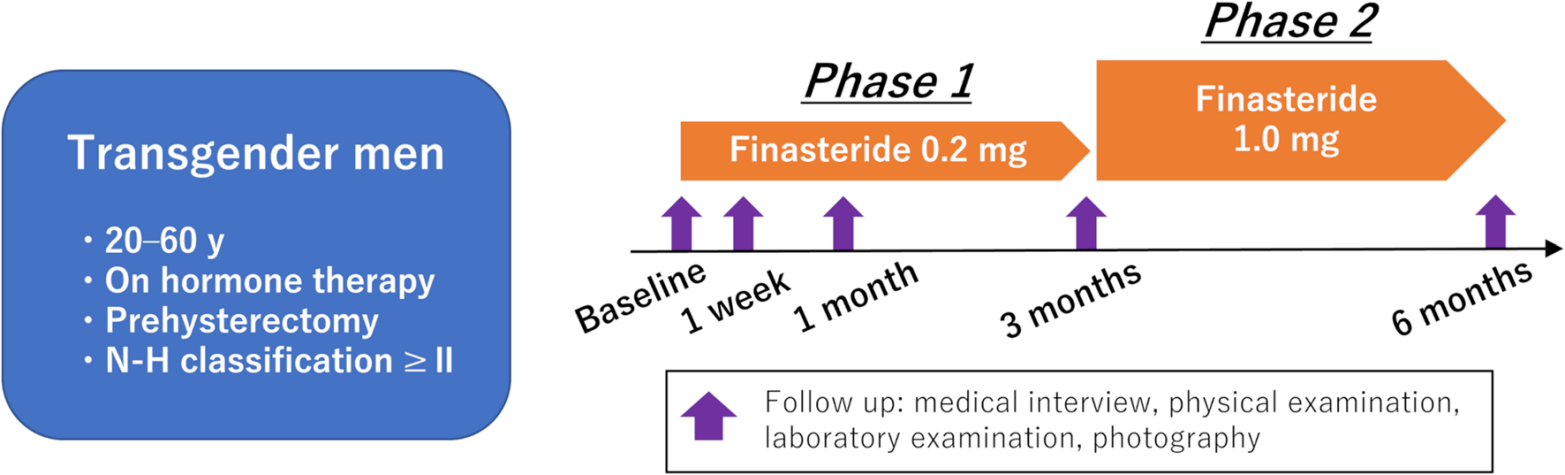
Inclusion criteria and study protocol

This study was designed as a single-arm, phase 1/2 trial. Figure 1 shows the study protocol. After eligibility assessment, the participants received 0.2 mg of finasteride orally once daily (after breakfast) for three consecutive months (phase 1). If patients completed 3 months of treatment without any significant adverse events, the dose was increased to 1.0 mg for another 3 months (phase 2). Treatment was discontinued in cases of adverse events of grade 2 or higher associated with finasteride, according to the Common Terminology Criteria for Adverse Events (CTCAE), version 5.0 [10]. At the time of the study design, the estimated annual number of transgender men with AGA at our institution was approximately ten, with an anticipated enrollment of five patients. Therefore, our target enrollment was five patients during the study period. Descriptive statistics were used to analyze the primary and secondary endpoints.

## Case presentation

A total of five participants were recruited, of whom three Asian transgender men (aged 44, 43, and 29 years) were ultimately included in the study (Table 1). All patients had a history of TRT use for more than 4 years but followed different TRT protocols. Patient A had a history of multiple medical conditions, including symptomatic epilepsy and Ménière’s disease, and was routinely taking multiple medications. Patients B and C had no current medical conditions requiring treatment and were not taking any medications.

**Table 1:** Baseline characteristics

	Patient A	Patient B	Patient C
Age, years	44	43	29
BW (kg), BMI (kg/m^2^)	62.6, 27.8	50.8, 20.3	84.5, 29.2
Duration of TRT, years	5	7	4
Testosterone preparation	Testosterone enanthate	Testosterone enanthate	Testosterone enanthate
Protocol	250 mg/4 weeks	125 mg/3 weeks	75 mg/2 weeks
Past medical history	Symptomatic epilepsy Sleep apnea Ménière’s disease Lymphadenitis Adenoid surgery	Lumbar disc herniation Kawasaki disease	None
Medication	Chlorpromazine hydrochloride, nitrazepam, etizolam, sodium valproate, foliamin, lacosamide, betahistine mesylate, mecobalamin, kallidinogenase, brotizolam, loxoprofen	None	None

*BW* body weight, *BMI* body mass index, *TRT* testosterone replacement therapy

Details of the primary endpoint results are provided in Table 2. All patients tolerated finasteride at a dose of 0.2 mg daily for the first 3 months, followed by 1.0 mg daily for an additional 3 months, without serious adverse events. The only adverse event potentially related to finasteride was loose stools in patient A. Patient A also developed gonococcal urethritis from contact with a sex partner, which was quickly cured with treatment at another hospital. The patient’s condition was not related to oral finasteride, and the patient was able to continue taking the medication. No adverse events were reported for patients B or C.

**Table 2:** Adverse events

	Patient A	Patient B	Patient C
Duration of finasteride	0.2 mg × 3 months 1.0 mg × 3 months	0.2 mg × 3 months 1.0 mg × 3 months	0.2 mg × 3 months 1.0 mg × 3 months
Adverse events, grade (associated with finasteride: ±)			
1 week	Seborrheic dermatitis, grade 1 (−)	None	None
1 month	Soft stools, grade 1 (±) Gonococcal urethritis, grade 2 (−)	None	None
3 months	None	None	None
6 months	Herpes labialis, grade 1 (−) Acute lumbago, grade 1 (−)	None	None

mo, month; wk, week

**Fig. 2:**
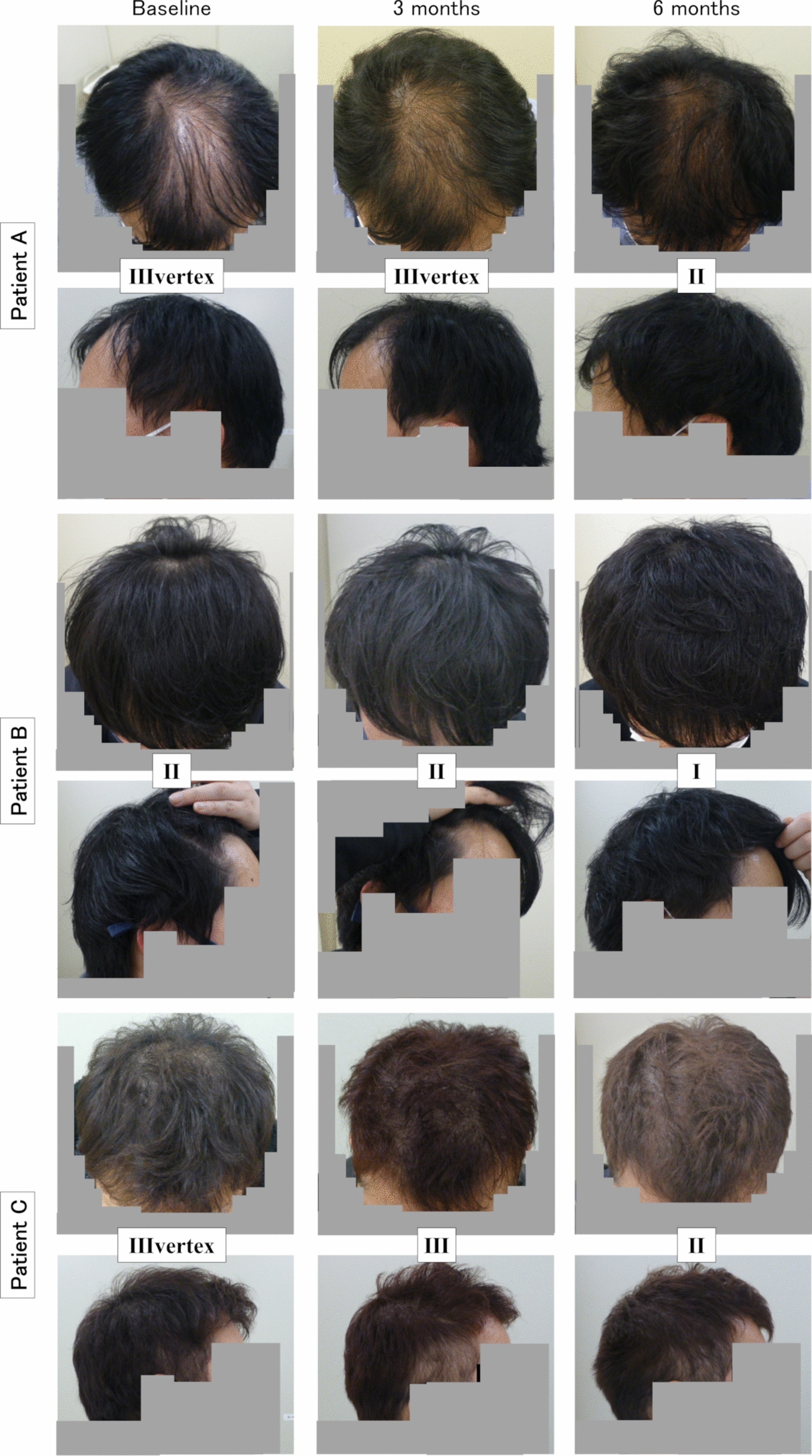
Changes in the Norwood‒Hamilton scale evaluated by photography of the forehead and vertex areas

To evaluate the efficacy of finasteride for AGA, photographs were taken from two angles at baseline, 3 months, and 6 months. The N–H scale was then assessed by the principal investigator (Fig. 2). Patients A and C had III vertex AGA according to the N–H scale, with baldness appearing in the vertex in addition to the forehead. Patient B had II AGA, with baldness limited to the forehead. After 3 months of finasteride at 0.2 mg, Patient C’s AGA in the vertex area improved, whereas patients A and B showed no Change. After an additional 3 months of finasteride at 1 mg, all patients showed an improvement in AGA of at least 1 stage. Additionally, all patients experienced a decrease in hair loss by the end of the observation period.

**Fig. 3:**
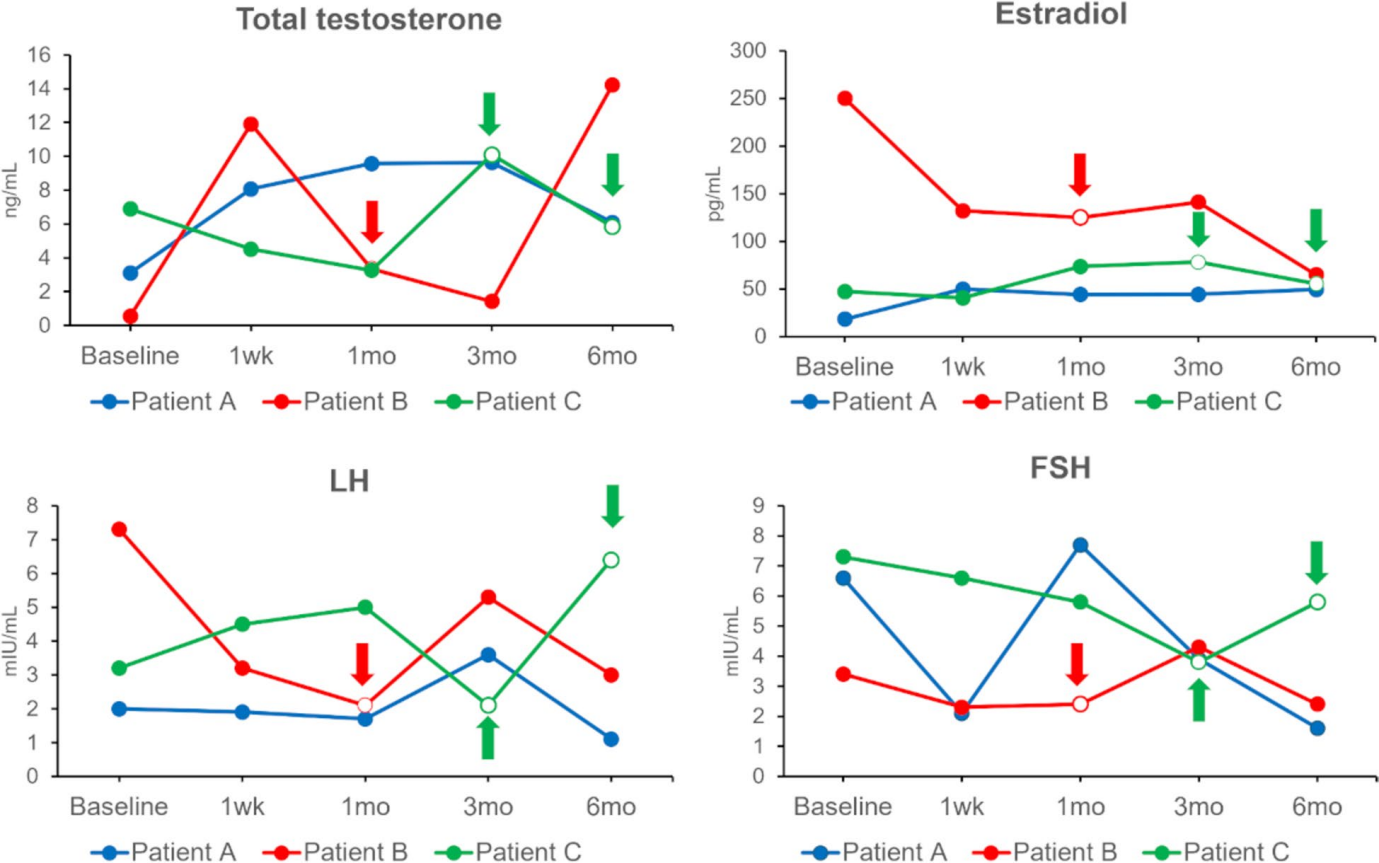
Changes in hormonal parameters

There was a slight overall increase in total testosterone despite periodic fluctuations (Fig. 3). In patient B, estradiol levels gradually decreased over the course of 6 months. In all patients, luteinizing hormone (LH) and follicle-stimulating hormone (FSH) levels exhibited greater variability; however, no consistent overall changes were observed. Notably, two of the three patients resumed menstruation, which had previously stopped during TRT treatment (as indicated by the arrows in Fig. 3).

All patients’ TRT protocols remained unchanged during the observation period. The other laboratory results are shown in Supplementary Figs. 1 and 2. Some variations were observed in renal function, liver function, and lipid profiles during the follow-up period, but no significant overall Changes were detected. Polycythemia, indicated by elevated red blood cells, hemoglobin, and hematocrit, gradually resolved after a peak at 1 month of finasteride administration.

Supplementary Fig. 3 shows that there were no significant changes in body weight, BMI, or body composition, including body fat percentage or lean body mass, over the study period. Two participants were obese at baseline (BMI ≥ 25 kg/m^2^).

## Discussion and conclusion

In this trial, we assessed the safety and efficacy of finasteride in transgender men with AGA. Three transgender men were observed for a total of 6 months. All patients were able to continue taking finasteride without experiencing any serious treatment-related adverse events. Finasteride was proven to be effective; all patients improved by at least 1 stage on the N–H scale. Notably, two of the three patients (66%) resumed menstruation, which had previously stopped because of their TRT protocol.

The average incidence of AGA in Japanese men of all ages is reportedly approximately 30% [11]. Heredity and androgen levels are known to play a role in its development, and its prevalence increases with age [12]. AGA in men is characterized by hair thinning in the frontal, frontotemporal, and vertex areas; women typically exhibit diffuse thinning of the vertex area with maintenance of the frontal hairline [9, 13]. Among transgender men, 17% of AGA diagnoses reportedly develop after 1 year of TRT, whereas 63% develop after 10 years of TRT [14, 15]. Similar to cisgender men, the frequency of AGA increases with longer hormone exposure and advancing age. However, it may be higher in transgender men than in cisgender men due to the greater influence of fluctuations in androgens and estrogen levels.

There are only two drugs approved by the United States Food and Drug Administration for AGA in cisgender men: topical minoxidil and oral finasteride [16]. A prospective study involving more than 3000 Japanese men with AGA demonstrated an efficacy rate of 87% for a 1-mg daily dose of finasteride, with a mean treatment duration of 459 days [7]. Reported adverse events included decreased libido, liver dysfunction, gynecomastia, and palpitations. Of particular concern is the potential negative impact on physical masculinization, as evidenced by decreased libido and gynecomastia. DHT binds to androgen receptors with greater affinity than testosterone and exerts stronger and more prolonged physiological effects. Therefore, taking finasteride may interfere with the development of masculine characteristics in transgender men.

Moreno-Arrones *et al*. evaluated finasteride use in transgender men with AGA classified as N–H stage III to IV [8]. All participants had undergone hysterectomy and oophorectomy and were on testosterone undecanoate for TRT. Of the ten patients, seven continued finasteride 1 mg daily for at least 1 year. Discontinuation occurred in one patient due to gastrointestinal symptoms and in two patients for economic reasons. No other adverse events were reported. Among the seven patients who continued treatment, all experienced at least a 1-stage improvement in their AGA classification. Despite differences in surgical history and hormone regimens, the adverse events and efficacy outcomes are comparable to those of the current trial.

In the present study, finasteride-associated interference with masculinization was evident through the resumption of menstruation. The participants with AGA tended to lower their TRT doses prior to the study in an attempt to avoid worsening AGA. In addition, two of the three were obese and did not necessarily have sufficient masculinization effects from TRT before starting finasteride. However, the fact that transgender men resumed menstruation after more than 4 years of treatment is remarkable. The mechanism by which testosterone administration results in the cessation of menstruation is not fully understood. Ovulation requires an increase in estrogen and an LH surge.

Previous studies provide insights into this phenomenon. Taub *et al*. conducted a prospective 12-week study examining ovulation in transgender men undergoing TRT [17]. They reported that testosterone may rapidly induce hypothalamic‒pituitary‒ovarian axis dysfunction and suppression in most individuals. Chan *et al*. demonstrated that estradiol levels decrease to normal male levels with TRT [18]. On the basis of insights from these studies, we propose the following reasons for the resumption of menstruation: first, treatment with finasteride, a 5-alpha reductase inhibitor, may lead to a reduction in DHT and a subsequent decrease in androgenic activity. Second, a relative increase in estrogen could occur, allowing for the restoration of the LH surge.

No significant Changes were noted in laboratory results or physical findings in our patients, except for the improvement in polycythemia after 1 month. While oral finasteride may increase testosterone levels, the current study used a short-acting formulation of testosterone enanthate, and the interval between the injection date and blood collection was not standardized. As a result, no clear trend was observed. Polycythemia is the most common adverse effect of TRT. Madsen reported that an increase in hematocrit may continue for up to 20 years after the initiation of TRT [19]. Therefore, it would be ideal if AGA improves, other masculinizing effects are not inhibited, and polycythemia is reduced. This may become possible in the future through adjustments to the TRT-finasteride protocols.

A 2023 review of AGA in TGD individuals recommended topical minoxidil, oral finasteride, and/or low-level laser light therapy as first-line treatment [20]. Although there are only a few reports evaluating the efficacy of topical minoxidil in transgender men, evidence exists for its effectiveness in both cisgender men and women, suggesting that it may also be effective for TGD people [16, 20]. Topical minoxidil is not believed to interact with TRT and is available as an over-the-counter drug in Japan. Therefore, we strongly recommend its use for transgender men with AGA. In contrast, while finasteride is considered a potential option, it may impair the masculinization driven by DHT [20, 21].

In the current trial, the finasteride protocol was divided into two phases to evaluate its safety and efficacy in a small number of patients. The dose-dense efficacy of finasteride was demonstrated. However, a report by Drake revealed that doses of finasteride as low as 0.2 mg per day maximally decrease DHT levels in both scalp skin and serum [22]. In the clinical setting, clinicians should be aware that continuing with 0.2 mg of finasteride may be sufficient to achieve an effective result.

While some transgender men may not be concerned about hair loss, most will find AGA troublesome. On the basis of the limited evidence from previous studies and the results of this trial, we suggest that clinicians provide a thorough explanation of the risks of AGA and take a family history of AGA before starting TRT. If AGA occurs after starting TRT, we recommend minoxidil at an early stage. If minoxidil is ineffective, 0.2 mg finasteride should be considered, but the protocol should be reassessed to ensure that it does not reduce the effectiveness of TRT.

This study has several limitations. First, the sample size was very small, with only three participants, partly due to a significant reduction in the number of outpatients during the oronavirus disease 2019 (COVID-19) pandemic. Second, DHT, a key androgen in AGA, could not be measured; instead, total testosterone, estradiol, LH, and FSH were assessed. Third, all participants received TRT via testosterone enanthate. In Japan, only short-acting testosterone enanthate is approved. Therefore, hormone levels of testosterone, estradiol, LH, and FSH are significantly influenced by the time elapsed since the last testosterone injection.

Nevertheless, this study is the only longitudinal prospective observational clinical trial evaluating the safety and efficacy of finasteride for transgender men who have not undergone hysterectomy-oophorectomy, making its findings both valuable and significant. This is especially important given the changing landscape in Japan, where gender-affirming surgery is no longer considered necessary for changing one’s legal gender.

This study suggests that finasteride is safe and effective for treating AGA in transgender men undergoing hormone therapy. However, concerns remain about its potential to interfere with the masculinizing effects of TRT. AGA in transgender men remains a significant issue, highlighting the need for further large-scale, long-term studies to better understand AGA management.

## Supplementary Information

Supplementary file 1.

Supplementary file 2.

Supplementary file 3.

## Data Availability

The trial results are publicly available through the Japanese Registry of Clinical Trials (jRCT); however, there are no plans to share individual participant data (IPDs).

## Abbreviations

TGD: Transgender and gender-diverse
TRT: Testosterone replacement therapy
AGA: Androgenetic alopecia
DHT: Dihydrotestosterone
N–H: Norwood–Hamilton
BMI: Body mass index
CTCAE: Common Terminology Criteria for Adverse Events
BW: Body weight
LH: Luteinizing hormone
FSH: Follicle-stimulating hormone
