# Supplementary figures and images for: The safety and efficacy of finasteride for transgender men with androgenetic alopecia: a case series
Source: J Med Case Rep. 2025 Sep 29;19:468. doi: 10.1186/s13256-025-05562-y (PMC12482827; doi:10.1186/s13256-025-05562-y)

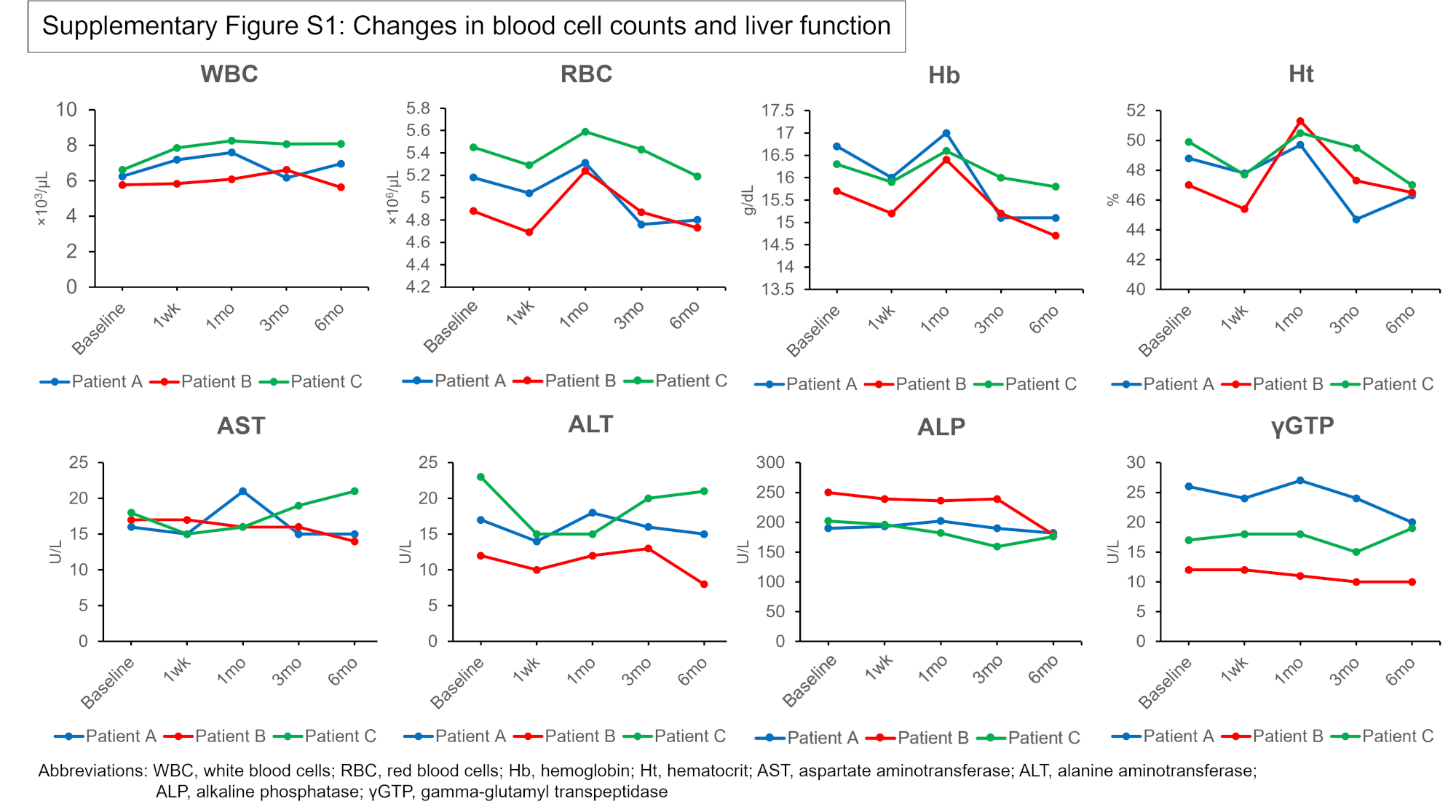

Supplement: Supplementary file 1 [file 13256_2025_5562_MOESM1_ESM.tif]

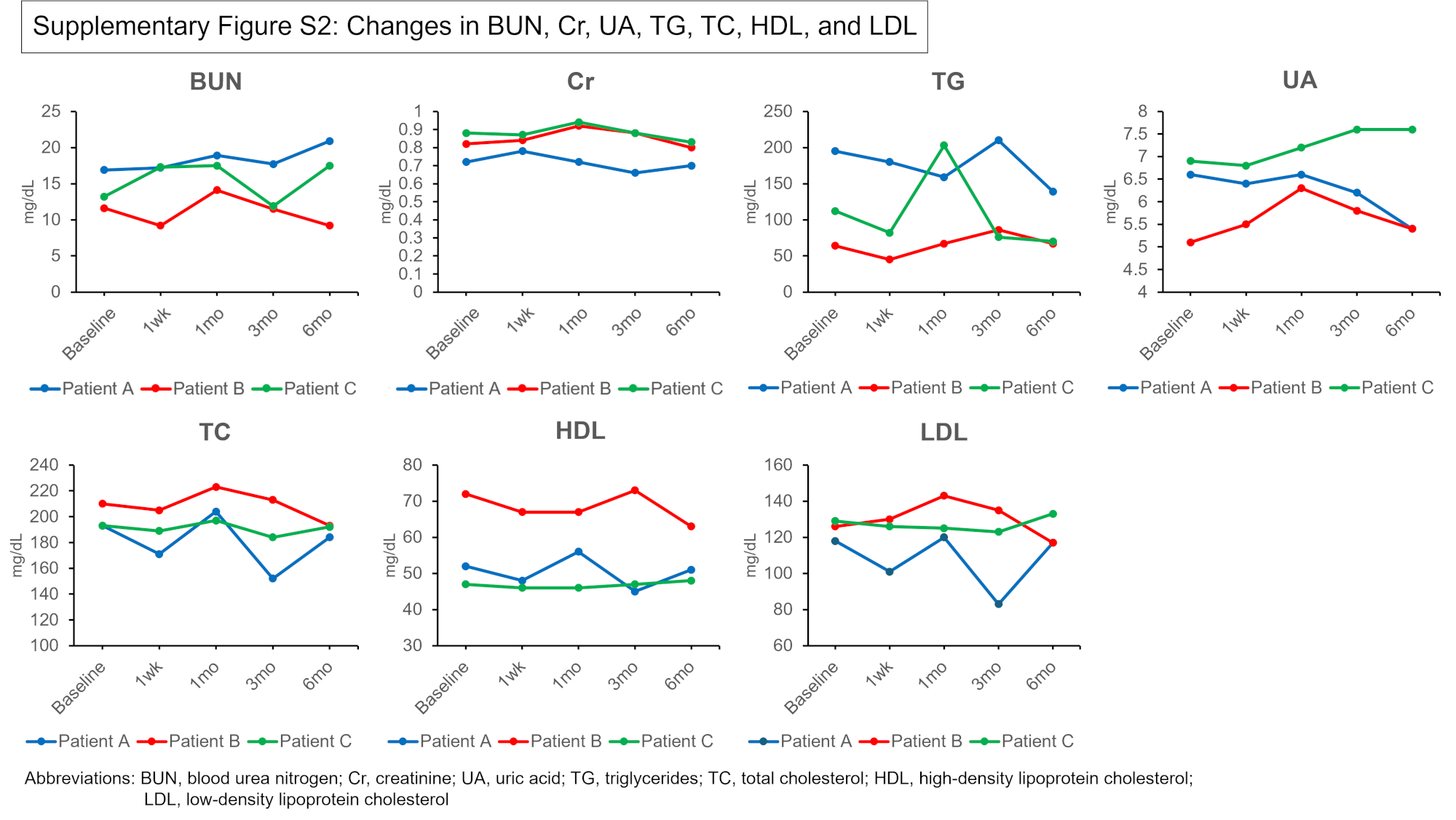

Supplement: Supplementary file 2 [file 13256_2025_5562_MOESM2_ESM.tif]

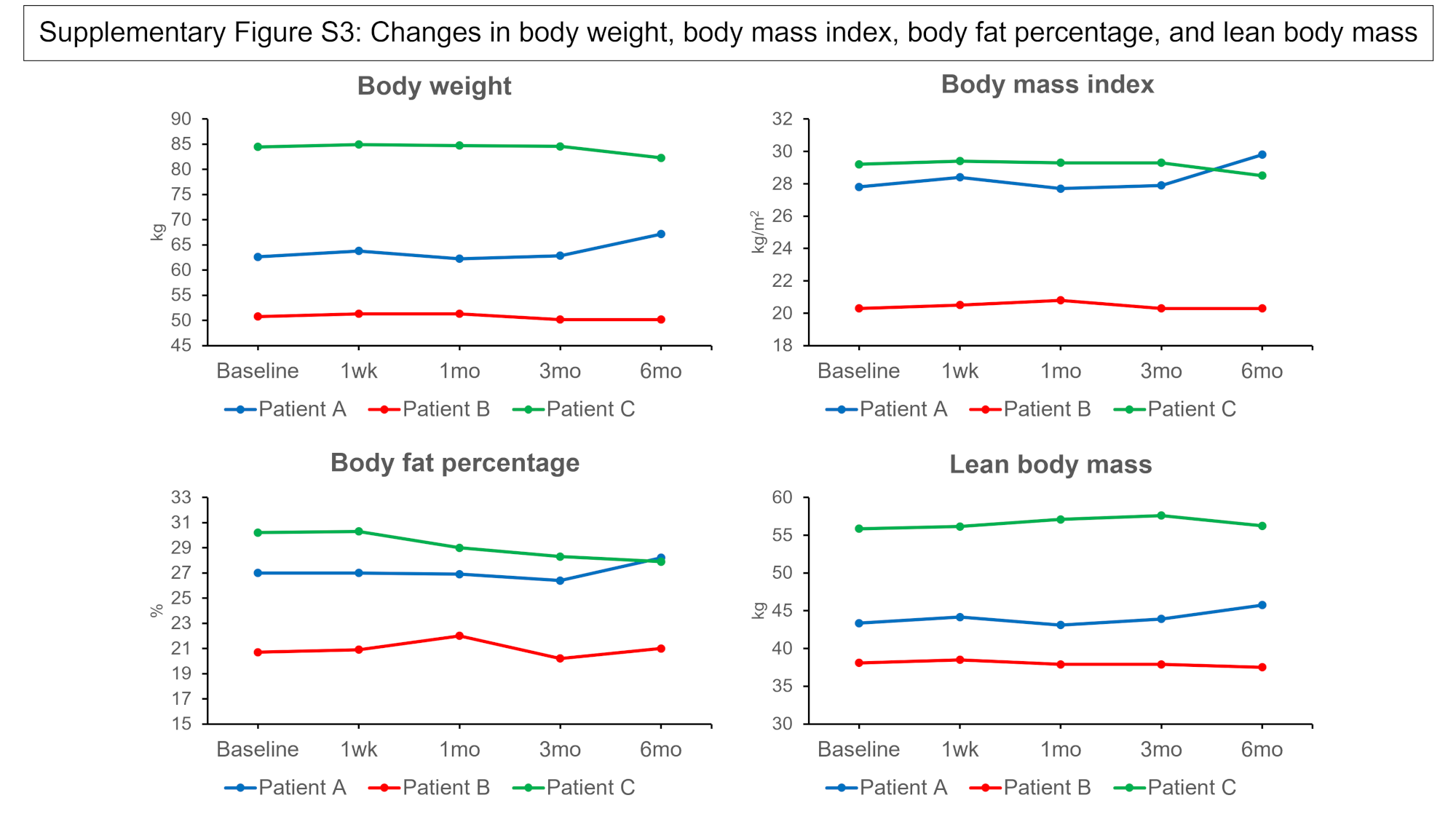

Supplement: Supplementary file 3 [file 13256_2025_5562_MOESM3_ESM.tif]
